# Childhood Health Condition, Midlife Lifestyle, and Later-life Mobility Function Trajectory

**DOI:** 10.1093/geroni/igaf122.3358

**Published:** 2025-12-31

**Authors:** Liu Huanran, Peiyi Lu, Vivian Lou

**Affiliations:** The University of Hong Kong, Hong Kong, China (People’s Republic); The University of Hong Kong, Hong Kong, China (People’s Republic); The University of Hong Kong, Hong Kong, China (People’s Republic)

## Abstract

Identifying individuals at risk of mobility decline early can enable timely interventions. Most studies focus on older age and lower limb mobility, overlooking overall mobility changes from middle age. Furthermore, the differential impact of early-life conditions on mobility trajectories across subgroups remains underexplored. Guided by cumulative advantage/disadvantage theory, this study examines how health-related early-life conditions is associated with mobility trajectories in Chinese middle-aged and older adults from a life course perspective. Data from the 2011- 2018 China Health and Retirement Longitudinal Study and the 2014 Life History Survey included 9,576 participants aged 45+. Mobility was assessed via a 7-item self-reported scale on any difficulty with mobility activities. Latent class trajectory modeling identified four trajectories: “High Mobility and Remain Stable” (50.31%), “Moderate Mobility and Deteriorate Swiftly” (24.96%), “Moderate Mobility and Fluctuate” (15.26%), and “Low Mobility and Decline Rapidly” (9.47%). Multinomial logistics regression model suggested individuals with poorer childhood health and a history of smoking in midlife were more likely to report declining or fluctuating trajectories. These findings highlight health-related early life conditions that can inform targeted interventions to promote mobility in older age, particularly those in disadvantaged childhood health condition and a history of midlife smoking behavior. Our research advances the cumulative advantage/disadvantage theory by illustrating how early-life conditions and midlife lifestyle factors collectively shape late-life mobility function trajectories from a comprehensive life course perspective.

